# Protocol for a co-design study for the development of a chronotherapeutic mobile health behaviour change intervention targeting light exposure among older adults

**DOI:** 10.12688/f1000research.157814.2

**Published:** 2025-07-25

**Authors:** Zahrah Alwi Alkaff, Resshaya Roobini Murukesu, Denz Del Villar, Julia Woerle, Manuel Spitschan

**Affiliations:** 1 TUMCREATE Limited, Singapore, Singapore; 2 TUM School of Medicine & Health, Department Health and Sport Sciences, Technical University of Munich, Munich, Bavaria, Germany; 3 TUM Institute for Advanced Study (TUM-IAS), Technical University of Munich, Garching, Germany; 4 Max Planck Research Group Translational Sensory & Circadian Neuroscience, Max Planck Institute for Biological Cybernetics, Tübingen, Germany

**Keywords:** Health Behaviour, Mobile Applications, mHealth, eHealth, Light, Light Exposure, Phototherapy, Patient Participation, Focus Groups

## Abstract

Through its wide-ranging effects on human physiology and behaviour, daily light exposure is an important environmental modulator of healthy ageing. Integrating mobile health (mHealth) technology with behaviour change strategies offers a promising approach to optimise light exposure and positively impact sleep, rest-wake cycles, cognitive function, and mood at scale. This study aims to develop the LightSPAN mHealth behaviour change intervention to optimise light exposure across the lifespan. Employing a co-design methodology, the study comprises two distinct workstreams. The first focuses on conceptualising the theoretical framework and implementation strategies through a comprehensive review of light exposure interventions, behaviour change theories, mHealth user personas, and recommendations for designing mHealth interventions for older adults. The second workstream centres on co-designing the intervention, involving consultation with community service providers and engagement with older adults at ageing community centres (≥60 years of age). Community service providers will be consulted through a focus group discussion (target n=5). Older adult participants (n=20) will engage in telephone interviews, focus group discussions and prototyping workshops to explore older adult participants’ characteristics, needs, preferences, and mHealth intervention design elements and co-design the LightSPAN mHealth behaviour change intervention. The insights generated in these co-design components will facilitate future implementation of our intervention.

## Background

Population ageing is a global megatrend. This is especially relevant to Singapore, where the proportion of older adults has nearly doubled, rising from 11.7% in 2013 to 19.1% in 2023. ^1^ Furthermore, Singapore has one of the world’s highest life expectancies and is the only country within Southeast Asia to surpass a life expectancy beyond 80 years. ^2^ However, living longer does not necessarily equate to living well. Unfavourable behavioural lifestyle practices have been shown to impact the quality of life, heighten susceptibility to chronic diseases, and shorten healthy life expectancy. ^3^

Additionally, the evidence suggests low levels of awareness regarding the benefits of adopting favourable behaviour modification, even in later stages of life. These changes can enhance self-reliance and self-management skills, improving functional capabilities and mitigating the risk of adverse health outcomes. ^4^ Hence, developing tailored lifestyle-oriented health interventions with embedded behaviour change elements into ageing-in-place strategies that align with daily living becomes increasingly essential to support healthy ageing, independence, and quality of life of older adults.

### Impact of light and healthy ageing

Light is a fundamental environmental stimulus impacting numerous aspects of human physiology and behaviour. In addition to enabling one to see and perceive the world, the non-image-forming (NIF) effects of light ^5^ include the alignment of the human internal biological clock to the environmental day/night cycle known as circadian rhythms, as well as the regulation of sleep patterns, alertness, mood, neuroendocrine activities, and cognitive functions. ^6^ These effects are mediated by specialised photoreceptors in the retina referred to as intrinsically photosensitive retinal ganglion cells (ipRGCs). ipRGCs are particularly sensitive to light and serve as conduits in conveying information about ambient light conditions to the brain. ^7^

With advancing age, significant changes in the visual system’s morphology and function occur progressively. These changes include a decrease in pupil diameter, a decrease in lens transmittance, presbyopia, the presence of vitreous floaters, and age-related macular degeneration. ^5, 8^ The decline in visual quality and function associated with ageing is characterised by decreased visual acuity, visual field sensitivity, contrast sensitivity, and increased dark adaptation threshold, attributed to age-related changes in retinal neural elements and ocular media. ^9^ These various changes lead to reduced admittance of light into the eye, consequently impacting the perception and processing of light. ^5^

In addition to the functional differences in visual ability, older adults face disruptions in their light exposure patterns due to decreased mobility, changes in sleep habits, and inadequate indoor lighting that can adversely affect well-being. ^6, 10, 11^ Older adults are more predisposed to facing challenges in maintaining consistent sleep patterns and quality than younger adults. ^12– 14^ With advancing age, the decline in the central master clock’s efficiency and a decrease in melatonin secretion lead to fragmented rest-activity and sleep-wake cycles. ^5, 15^ The interplay of two systems, the sleep-wake homeostatic drive and the internal circadian clock, tends to be diminished in older adults, affecting the regulation of sleep-wake cycles. ^14, 16^

In addition to circadian entrainment and sleep, light influences cognitive function and mood through non-visual pathways. The limbic structures in the brain, namely the hippocampus and amygdala, activated by retinal light exposure, regulate mood and cognitive function. ^17^ Moreover, the circadian and homeostatic sleep-wake regulating systems interact differently to affect human cognitive performance and mood. ^18^ Exposure to bright light, particularly natural daylight, enhances alertness, mood, and cognitive performance. ^7, 19^ Conversely, aberrant light exposure can lead to mood disturbances, fatigue, and impaired cognitive function. ^20, 21^ Crucially, increased light exposure at night has been associated with a heightened risk of severe mental health conditions, including major depressive disorder, anxiety disorders, post-traumatic stress disorder, and self-harming behaviours. ^22^

Lifestyle choices, social aspects, and environmental factors influence light exposure patterns. ^23^ Thus, reduced admittance of light through the ageing retina, coupled with modifications in self-selected light exposure, lead to dysregulation in circadian rhythms, sleep-wake cycles, timing and quality of sleep, alterations in mood, behaviour, cognitive function, and increased susceptibility to morbidity and mortality in older adults. ^24, 25^ Ultimately, this leads to compromised well-being, alteration of the healthy ageing trajectory, diminished resilience, and premature mortality.

To address these age-related challenges, therapeutic approaches targeting the alignment of circadian rhythms may have protective benefits in preventing or mitigating age-related diseases. ^26^ Evidence-based recommendations for healthy light exposure throughout the day have been developed for healthy adults. ^27^ These recommendations highlight the importance of appropriate light exposure timing and the key concept of ‘bright days and dark nights’ to reap the benefits of light exposure ^27^ but do not provide practical recommendations for realising these criterion levels. Hence, understanding the nuanced relationship between ageing and light exposure is essential for promoting healthy ageing trajectories and enhancing the quality of life for older adults.

### Optimising light exposure for older adults through behaviour change via mHealth

Given the interplay between ageing, light exposure, and physiological outcomes, there is a pressing need for targeted interventions to optimise light environments to promote healthy ageing. Tailored interventions incorporating principles of chronobiology and light therapy, such as bright light therapy, environmental modifications, and timed exposure to daylight, have shown promise in improving sleep quality, cognitive function, and mood in older adults. ^8, 28– 32^ While traditionally viewed as a passive environmental factor, light exposure is now seen as a dynamic behaviour, encompassing choices regarding various light sources and exposure times. ^33^ Understanding light exposure through a behavioural lens can shape light exposure-related behaviours by empowering individuals to make informed decisions regarding their light exposure and inform targeted interventions. ^7^

Digital technologies have emerged as powerful tools in public health for promoting healthy lifestyles, with mobile health (mHealth) interventions standing out as promising avenues for facilitating behaviour change. ^34– 36^ Leveraging the increasing technical sophistication of mobile phones, mHealth offers accessible and scalable platforms for delivering targeted interventions. ^37, 38^ Pairing mHealth technologies with activity prescription has demonstrated favourable outcomes as a health promotion initiative aimed at preventing and mitigating the risk of lifestyle-related health outcomes. ^39^ Recent systematic reviews have highlighted mHealth as an effective tool for promoting health behaviours, managing chronic conditions, and improving medication adherence. ^40– 42^ In Singapore, stakeholders have increasingly leveraged digital platforms to promote healthy behaviours. ^43^ However, despite older adults’ interest in these interventions, challenges such as low confidence and anxiety regarding technology use, negative attitudes toward digital tools, trust and privacy concerns, limited digital literacy, and age-related limitations hinder user engagement. ^44– 48^ These challenges necessitate fine-tuned implementation strategies to optimise the accessibility, acceptability, and adaptability of mHealth interventions targeting ageing societies. Thus, mHealth interventions must be tailored to meet older adults’ needs and preferences, incorporating appropriate behaviour change strategies and undergoing iterative refinement based on user feedback and real-world data to enhance effectiveness and user satisfaction. ^38, 49^

### Co-design approach for the development of healthy ageing mHealth interventions

The success of any intervention hinges on users’ adoption, adherence, and sustained engagement, which depends on technology’s capacity to overcome barriers and adapt to user needs and real-world contexts. ^50^ The successful implementation of technology for ageing-in-place relies on prioritising the needs and wishes of older adults throughout the development and deployment phases, ensuring acceptance of the technology, delivering tangible benefits, and establishing favourable conditions conducive to using technology. ^51^ Lived experiences as older individuals have been shown to offer invaluable insights often overlooked in technology development. ^52^ Nonetheless, older adults have expressed an eagerness to learn and a willingness to engage in co-design initiatives. ^52^

Co-design is a collaborative approach where stakeholders such as users and community members are involved in the development and design process of products or interventions. This process is recognised for its ability to better address the population needs and yield better outcomes. ^53– 55^ It aligns with current policies, care practices, and the growing body of evidence highlighting the importance of engagement and co-design in health promotion interventions and digital solutions. ^53, 56– 58^ Existing literature has established the utility of co-design, including a study that identified potential strategies and design alterations for a smartphone app through co-design workshops with people living with dementia and their care partners. ^59^ Overall, studies using a co-design approach for technologies in health management, fitness, or in-home wellness have recognised the importance of including older adults as co-designers. ^60– 62^ Through sharing knowledge, experiences, and preferences, co-design optimises evidence-based interventions according to stakeholders’ priorities, facilitating feasibility and sustainability. ^63^

## The LightSPAN Project

### LightSPAN for healthy ageing: Optimizing light exposure via an mHealth behaviour change intervention in Singapore

The LightSPAN project in Singapore tackles an essential aspect of healthy ageing by recognising the significant role of light exposure in regulating human health and quality of life. Amidst Singapore’s urbanisation, high population density, tropical climate, and growing elderly population, older adults face additional challenges compounded by age-related pathologies. ^43, 64^ These include restricted access to daylight, heightened exposure to artificial lighting, discomfort from the region’s heat and humidity, and the prevalent issue of light pollution. ^64, 65^

To address these challenges, the LightSPAN project is embarking on developing and testing an mHealth behaviour change intervention as an ageing-in-place strategy to empower older adults in Singapore, supporting them in adopting light-friendly behaviours that can positively influence their health. The intervention will be delivered through a novel app, the LightSPAN mHealth app, which tracks light exposure using a light sensor and employs various behaviour-change strategies to optimise light exposure patterns, with the ultimate goal of promoting the potentially protective effects of light exposure on circadian rhythms, sleep quality, mood regulation, alertness, and cognitive function in ageing individuals. Through this innovative approach, LightSPAN seeks to provide valuable insights into the potential efficacy of a lifestyle-oriented intervention using smartphone-based app technology in promoting well-being among the older adult population in Singapore. This protocol concerns the co-design stage of the LightSPAN project, which will be followed by a pilot and feasibility trial, and a randomized controlled trial (RCT).

### The current study: Developing the LightSPAN mHealth app to optimise light exposure for older adults

The current study presents the protocol outlining the formative steps of LightSPAN’s smartphone-based behaviour change intervention designed to optimise light exposure across the lifespan. This work is a precursor to a pilot and feasibility trial, and a randomised controlled trial, which will evaluate the intervention’s effects on various metrics, including changes over time in light exposure, sleep, mood and well-being, cognitive assessment, and biometric health measurements. A co-design approach will be employed to develop an effective mHealth intervention tailored to our target users, involving active collaboration between the researchers and older adult participants. Building upon the recognised importance of light as an environmental facilitator of healthy ageing, this study aims to develop an mHealth behaviour change intervention to optimise light exposure among older adults in Singapore via a novel mHealth application. Specifically, this study aims to:

1. Conduct a literature search on light exposure interventions, behaviour change theories, mHealth user personas, and recommendations for designing mHealth interventions for older adults to conceptualise the theoretical framework and implementation strategies.
2. Consult with community service providers through focus group discussions (FGD) to gather insights and feedback on the proposed intervention plan.
3. Undertake a telephone interview to understand older adults’ current practices and preferences related to health, light exposure, and technology.
4. Conduct focus group discussions with older adults to explore their perspectives, needs, and preferences regarding light exposure, motivations for behaviour changes, and acceptance of mHealth interventions and gather feedback on the LightSPAN mHealth intervention plan.
5. Conduct a prototyping workshop to present the prototype of the LightSPAN mHealth app, aiming to collect feedback for its iterative development and refinement.

## Methods

### Study design

**Figure 1.**
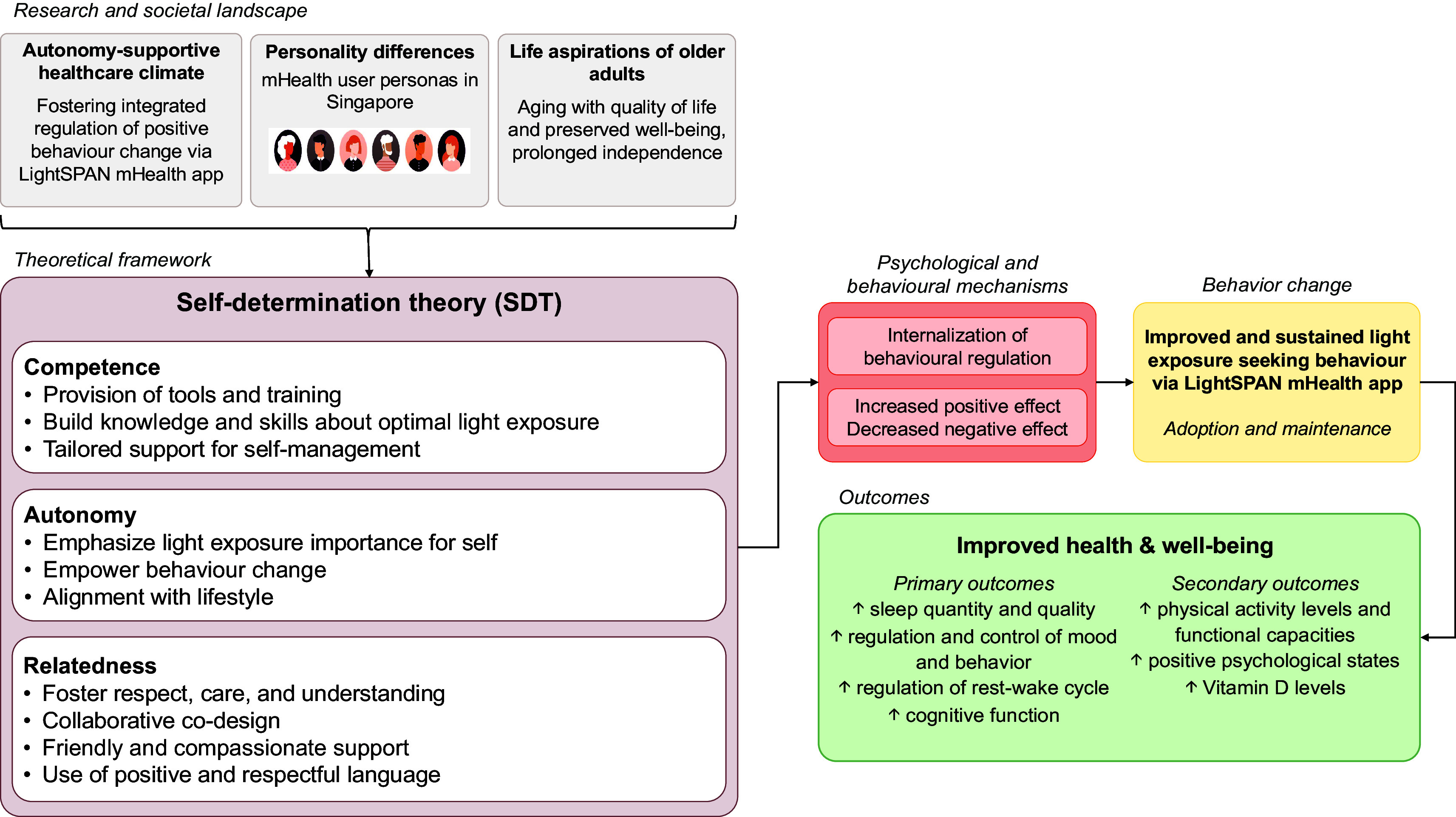
Study flow diagram for the LightSPAN Co-design protocol.

The present co-design will adopt a mixed-methods approach structured across two main workstreams. The study flow chart depicted below in Figure 1 outlines the co-design protocol, describing the progression from one workstream to another and the content explored at each stage.

• **Workstream 1:** Conceptualization of the theoretical framework and implementation strategies
  ○ 1a: Overview of Reviews
  ○ 1b: Examination of mHealth personas
  ○ 1c: Self-Determination Theory (SDT) behaviour change framework
  ○ 1d: Recommendations for designing mHealth interventions for older adults
• **Workstream 2:** Co-design of the mHealth intervention
  ○ 2a: Consultation with community service providers
  ○ 2b: Engagement with older adult participants
    ▪ Telephone interview
    ▪ Focus Group Discussion
    ▪ Prototyping workshop

### Research project team

The research project team is comprised of a research team including a circadian and visual neuroscientist (MS, PhD, male), an ageing health scientist (RRM, PhD, female), both with more than five years of experience in human research, as well as a research associate with training in cognitive neuroscience (ZAA, MSc, female), a research software engineer (DDV, BSc, male), and a clinical trial coordinator (RBS, BSc, female), all with less than five years of experience in human research.

During the focus group discussion and prototyping workshop, the team will be assigned roles to support smooth running of the sessions, mainly a moderator, facilitator, and notetaker. The moderator will lead the discussion, pose questions, and encourage participation. The facilitator will manage session logistics and step in to support the moderator or participants as needed to maintain session flow. The notetaker will document key points using a structured template based on prior guidelines. ^66^

### Guiding principles

The current study advocates for the inclusion of an additional construct focused on behaviour change to push beyond the envelope of merely developing an effective mHealth intervention but also one that is an integrated mHealth solution tailored to meet the unique needs and preferences of its users and creates sustained, long-term, positive health behaviour practice among older adults. In line with recommendations for person-based approaches to intervention development, we formulated guiding principles for the intervention design. These serve as a reference in the planning and development stages. ^67^

Comprising two essential elements, they include intervention design objectives and key features crucial for achieving these aims. The first intervention design objective is to educate older adults on the importance of light exposure and its associated behaviours by developing informative content and materials and providing tools and resources that allow participants to make informed decisions about their light exposure habits, aligning interventions with their preferences and lifestyles. The second intervention design objective is to help older adults optimise their light exposure to support their health and well-being through mHealth by employing behaviour change techniques, including goal setting, self-monitoring, and feedback to encourage sustained engagement with the intervention by fostering autonomy, competence, and relatedness and adoption of healthy light exposure practices.

Our approach’s key components are empowering participants to enhance their well-being through optimised light exposure, creating lifestyle-compatible practices, promoting self-efficacy and positive affect through encouraging and rewarding achievable goals, and providing support and strategies to overcome barriers. To facilitate this, tailored interventions must be delivered appropriately for older adults, considering the population’s cultural and circumstantial context, for behaviour change to be effectively harmonised within individuals’ lifetimes. ^68^

A core concept in the delivery of the intervention, the deployment of an app, allows for the scalability of the approach.

**Workstream 1: Conceptualization of the theoretical framework and implementation strategies**

Aim: To conduct a literature search on light exposure intervention, mHealth user personas, behaviour change framework, and recommendations for designing mHealth interventions for older adults to conceptualise the theoretical framework and implementation strategies.

The first workstream is the cornerstone of the intervention development, laying the foundation for the subsequent workstreams. This involves carefully reviewing the literature to synthesise current evidence, ensuring it is informed by evidence-based practice. The research efforts within this workstream encompass four parts as follows.

**Workstream 1a: Overview of Reviews**

In Workstream 1a, we aim to address the research question: What are the effects of ocular light exposure on sleep, circadian rhythms, rest-activity cycles, mood, and cognitive function in older adults aged 60 years and above?

A Cochrane Overview of Reviews will be undertaken to identify existing systematic reviews on light as a lifestyle intervention to enhance the health and well-being of older adults. This Overview of Reviews aims to comprehensively understand the efficacy and potential benefits of ocular light exposure in this demographic. Following the proposal approval of this Overview of Reviews from the Cochrane Database of Systematic Reviews, the protocol and review will follow the Cochrane Review guidelines. Preliminarily, we have identified a collection of existing systematic reviews that provide insight into ocular light exposure and health outcomes on older adults, specifically on sleep, circadian rhythms, rest-activity cycles, mood, and cognitive function. ^30, 69– 80^ The findings in this Overview of Reviews will provide systematised scientific knowledge on how light exposure can support healthy ageing and develop an understanding of target light levels for optimal beneficial effects.

**Workstream 1b: Examination of mHealth user personas**

Workstream 1b aims to address the following research question: What are the distinct personas and associated characteristics of older adults aged 60 years and above in Singapore concerning their engagement with mHealth solutions?

To understand the diverse needs, preferences, and behaviours of older adults in Singapore regarding mHealth solutions, we conducted a purposive search for persona papers related to mHealth users, focusing on older adults in Singapore. This involved a thorough exploration to identify relevant studies and comprehensively review them to extract insights into the needs and preferences of older adults in Singapore concerning mHealth solutions. The search led to a study by Haldane et al. ^81^ that identified five personas through 20 in-depth interviews and 100 survey responses: The Quiet Analog, The Busy Grandparent, The Socializer, The Newly Diagnosed, and The Hard-to-Reach. These personas delineate vital characteristics such as healthcare access, medication adherence, mobile phone technology usage, and interest in mHealth. By benchmarking this study, our goal is to enrich our understanding of user behaviours, preferences and needs within the context of our research.

The five personas highlight variability across various dimensions, including lifestyle, social factors, and mobile technology usage and needs. The personas also reveal behavioural variations in adherence among older adults and usability issues, illustrating how users interact with their phones in specific ways. Thus, to support uptake and adherence and effectively cater to the different personas, the intervention development must address varying levels of familiarity with mHealth, such as incorporating introductory sessions for those less familiar and having trusted providers facilitate the process. The personas also elucidated why certain groups may be hesitant to adopt mHealth interventions, thereby prompting exploring strategies to gain their buy-in or developing alternative or supporting measures to support them effectively. Lastly, a takeaway from the personas is that they can help facilitate focused discussions during design by prompting discourse on how each persona may respond to tasks, prompts, or calls to action. These personas can be valuable tools, providing a tangible framework to conceptualise and empathise with our target demographic. Integrating insights from these personas ensures that our solutions are finely attuned to the diverse needs and preferences of older adults in Singapore, thereby enhancing the likelihood of successful uptake and adherence to our mHealth intervention.

**Workstream 1c: Self-Determination Theory Behaviour Change Framework**

In Workstream 1c, we seek to address the following research question: “What behaviour change model can be applied to optimise light exposure among older adults and promote sustainable behaviour change in this population?”

Following the evaluation of various behaviour change strategies such as the Health Belief Model, Theory of Planned Behaviour, and The Transtheoretical Mode, the Self-Determination Theory (SDT) stands out as a prominent framework for understanding and promoting behaviour change in older adults, given its foundation in motivation and well-being, its wide use, along with the growing evidence of efficacy. ^82, 83^ Additionally, the SDT has particular relevance and effectiveness in addressing older adults. Its emphasis on autonomy and empowerment aligns well with the needs and preferences of this population, making it a suitable framework for promoting healthy behaviours and enhancing well-being in older age. ^84^ The SDT was thus selected as the theoretical framework to guide the development of intervention and behaviour change strategies.

The SDT posits that human motivation is driven by three basic psychological needs: autonomy, competence, and relatedness. Individuals are intrinsically motivated to engage in activities fulfilling these needs, leading to greater persistence, satisfaction, and well-being. ^85^ To exemplify, a study by Teas et al. posits that merely encouraging and partaking in physical activity was insufficient to improve well-being in older adults. It was emphasised that these activities must also fulfil their psychological needs. ^86^ Extensive research has shown the effectiveness of interventions based on the SDT in health promotion, education, and workplace performance. ^87– 91^ Additionally, resources are available that elucidate the principles of the SDT and provide guidance for its application within the context of health, promoting and encouraging its utilisation. ^67, 92^

**Figure 2.**
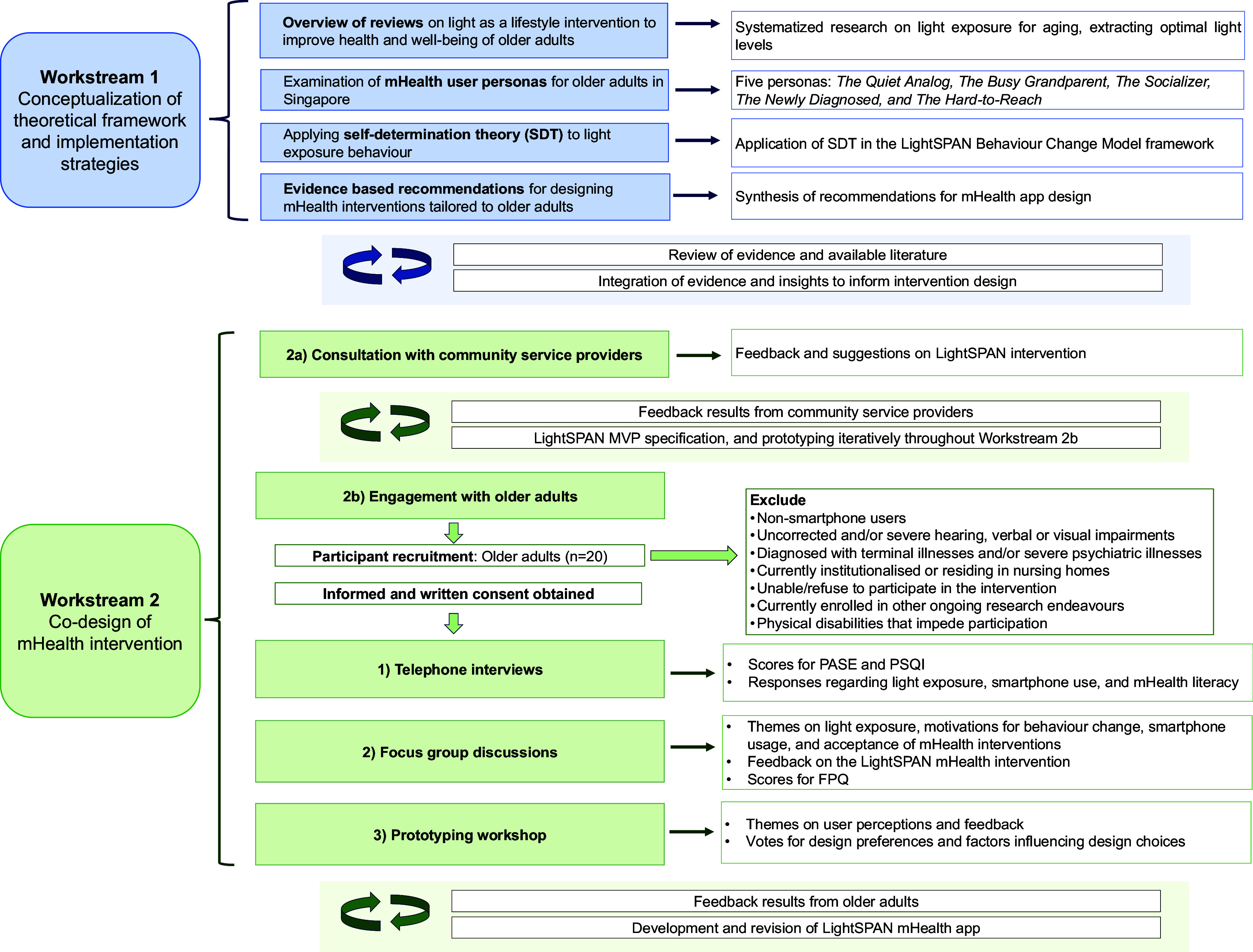
Application of the Self-determination theory (SDT) in the LightSPAN project Behaviour Change Model framework, serving as a working model for this project.

When applied to the context of light exposure, the SDT offers valuable insights into the motivational factors that can drive individuals’ light-related behaviours. The LightSPAN Behaviour Change Model framework is formulated based on the insights derived from the SDT, as shown in Figure 2. The framework outlines the concepts by which we seek to foster autonomy, competence, and relatedness among participants, facilitating behaviour change in the context of light exposure.

In this framework, autonomy emphasises empowering individuals to make informed choices about their light exposure. This involves highlighting the importance of light for health and well-being and enabling participants to align their behaviour changes with their personal lifestyles and routines. By giving them control over implementing these changes, the intervention fosters a sense of ownership, making it more likely that participants will sustain their behaviour long-term.

Competence focuses on providing participants with the tools, education, and support needed to manage their light exposure effectively. This includes practical resources like light sensors and the LightSPAN mHealth app, which help participants track and adjust their exposure. The intervention also focuses on building knowledge, raising awareness of the benefits of optimal light exposure and equipping participants with the necessary skills to apply this knowledge. Additionally, tailored support ensures they have guidance to manage their progress confidently. This creates a sense of achievement and mastery as participants learn and apply new skills.

Relatedness is addressed by fostering a sense of care, respect, and connection between participants and the intervention team. Through collaborative co-design, participants’ feedback and preferences are considered, making them feel valued and understood. The language and interactions used are friendly and compassionate, encouraging a positive and respectful atmosphere that supports participant engagement. This principle also ensures that the participants feel connected to the broader goals of the intervention and the community.

The intervention will incorporate a series of techniques—self-monitoring, goal setting, feedback, and education—that are integrated to align with SDT principles of autonomy, competence, and relatedness. These techniques have been proposed to engage participants with technology and produce the desired behaviours. They have been shown to be effective in digital interventions across various studies, ^93– 97^ particularly in supporting self-efficacy and positively impacting behaviour. ^98, 99^ These techniques will work together within the LightSPAN mHealth app to create a cohesive and supportive intervention experience:

▪ Self-monitoring empowers participants by giving them real-time data on their behaviour, allowing them to take control of their light exposure and make adjustments.
▪ Goal setting helps participants personalise objectives, breaking changes into achievable steps that foster confidence and a sense of progress.
▪ Feedback reinforces this progress by providing supportive insights and creating a caring environment that motivates continued engagement.
▪ Education underpins these techniques, providing participants with the knowledge and skills needed to make informed decisions.

Together, these techniques foster a sense of empowerment, mastery, and connection, motivating participants to sustain long-term change by aligning their actions with their personal goals and feeling supported throughout the process.

**Workstream 1d: Evidence-based recommendations for designing mHealth Interventions for older adults**

Workstream 1d addresses the following research question: “What evidence-based recommendations can guide the design and implementation of mHealth interventions tailored to older adults?”

To ensure the efficacy of our mHealth interventions tailored to older adults, we explored mHealth studies and established best practices. This step aims to identify strategies that have effectively leveraged technology to address older adults’ health-related concerns. Through a review of the evidence, we searched for studies on digital and mHealth interventions and co-design studies involving older adults. From these studies, we extracted evidence-based recommendations to guide the design and implementation of our mHealth interventions. These recommendations were synthesised to inform our app’s development and design process, ensuring it aligns with practices conducive to promoting health among older adults.

The evidence-based recommendations will be infused into the intervention through these five broad categories: app features, engagement strategies, development methodologies, interface design, and support mechanisms, providing a structured framework to guide the design and implementation of the app suitable for older adults.

**Integrating insights**

After thorough reviews and analyses of the evidence and available literature, we integrate our insights to inform the initial development phase and systematically guide subsequent steps in our project. This process informs the intervention design, focusing on developing the LightSPAN mHealth app developed following the additional work in Workstream 2. We aim to develop and present an evidence-based prototype of the smartphone application that aligns with the objectives and framework of the LightSPAN mHealth intervention, tailored to the needs of our target audience, and positioned to effectively promote healthy ageing.

**Workstream 2: Co-design of the mHealth intervention**

Workstream 2 centres on the collaborative design process of the mHealth intervention. It involves a comprehensive exploration of participant characteristics and needs alongside the design elements of mHealth interventions. To achieve this, we employ a multi-pronged approach consisting of several data collection methods. The first group comprises community service providers to seek their experience and knowledge to provide their insights and feedback on the proposed intervention plan.

The second group comprises community-dwelling older adult participants who partake in telephone interviews, FGDs, and prototyping workshops.

As part of the development process, we will outline a Minimum Viable Product (MVP) of the LightSPAN mHealth app, specifying the core functionalities of the app. This MVP will serve as a baseline for the early development of the prototype, which will be iteratively built and refined based on feedback gathered throughout the co-design process. This version will be refined through focus group discussions, prototyping workshops, and feedback from participants.

These methodologies enable collaborative development of the LightSPAN intervention with the study team, ensuring the active involvement of older adults in shaping the app according to their needs and preferences.

**Workstream 2a: Consultation with community service providers**

The consultation with community service providers aims to gather insights and feedback on the proposed intervention plan tailored for older adults. Invitations will be extended to community service providers working at Lions Befrienders (LB), a social service agency in Singapore dedicated to providing care and support for older adults. ^100^ We will recruit from a pool of community service providers, comprising individuals in roles such as programme staff, centre managers, administrative staff, and those involved in service delivery, community engagement, or innovation, based on convenience, with no specific inclusion or exclusion criteria (except for ≥18 years of age). The consultation will be conducted as a focus group discussion, guided by a pre-developed topic guide. During the consultation, the research team will present the proposed intervention plan and seek feedback, suggestions, and insights regarding the plan’s relevance, feasibility, and potential impact within the community setting. The session will cover potential barriers and facilitators to implementation, strategies for engagement and recruitment of older adults, and any additional considerations. Feedback collected during the consultations will help identify areas for improvement and refinement of the intervention.

**Workstream 2b: Engagement with older adult participants**

*Study sample*

The study will involve community-dwelling older adults in Singapore recruited via purposive sampling. Participants will be recruited from two Active Ageing Centres (AAC) with the support of Lions Befrienders. The AACs are a drop-in social, recreational hub for older adults in the community in various neighbourhoods across Singapore. Upon recruitment, participants will receive a comprehensive information package containing an invitation letter, participant information sheet, consent form, and contact details of the research team. The target sample size for the co-design study is 20 older adult participants, guided by previous co-design studies. ^101, 102^ This sample will be divided into four groups of five participants matched in sex and age for the focus group discussions and subsequent prototyping workshop sessions. The participants and researchers will have no relationship prior to the study. Eligible participants will be recruited according to the inclusion and exclusion criteria, including smartphone use and demographic and mental and physical health parameters. The specific inclusion and exclusion criteria are outlined in Tables 1 and 2 below. 

**Table 1.** Inclusion criteria for participants.

Domain	Criterion	Assessment method
**Age**	≥60 years of age	Self-report
**Community dwelling**	Residing in Singapore, not currently institutionalized or residing in nursing homes	Self-report
**Mobility**	Capable of independent walking with or without assistive devices	Self-report
**Functional Independence**	Capable of functional independence	Lawton Instrument for Activities of Daily Living (IADL). Score > 8 for females, > 5 for males
**Language**	Proficiency in communication, reading, and writing in English	Self-report
**Smartphone use**	Own smartphones	Self-report
**Participation**	Not currently enrolled in other on-going research endeavors	Self-report

**Table 2.** Exclusion criteria for participants.

Domain	Criterion	Assessment method
**Cognition**	Cognitive impairment	Montreal Cognitive Assessment (MoCA), score ≤ 26
**Mental health**	Depressive symptoms	Geriatric depression scale (GDS) short form, score > 5
**Physical disabilities**	Significant physical impairment impacting daily activities	Self-report
**Health status**	Current diagnosis that interferes with daily function, and presence of severe terminal illness and/or psychiatric conditions	Self-reported with Self-Administered Comorbidity Measure (SCM)
**Visual health**	Visual impairment, diagnosis of eye diseases	Self-report
**Auditory health**	Uncorrected hearing impairment	Self-report

### Setting

The data collection sessions will take place at the AACs designated by LB and will utilize dedicated discussion rooms. These rooms will be set up to ensure a conducive environment, prioritizing comfort, privacy, and minimal disruptions, ensuring optimal conditions for engagement with older adults. ^103^

## Screening

### Background information form

At screening, demographic information will be obtained, including age, sex, ethnicity, marital status, education level, employment status, housing type, living arrangement, socioeconomic status, and contact information. Participants will also be screened for functional mobility, communication abilities, smartphone ownership, and health status.

### Health variables

Participant’s overall health will be assessed to determine health status, cognitive function, mental health, and ocular health. Table 3 presents the screening measures that will be administered for participant recruitment.

**Table 3.** Screening measures for study participants.

Construct	Instrument	Administration and instrument details	Outcome
**Functional Independence**	Lawton Instrument for Activities of Daily Living (IADL), developed by Ref. 104	Participants will complete the Lawton IADL to evaluate an individual’s self-reported ability to perform instrumental activities of daily living. The scale assesses a person’s functional independence in more complex activities that are necessary for independent living in the community such as managing finances, preparing meals, shopping, doing housework, using transportation, handling medications, and using the telephone. A score of > 8 for females, > 5 for males indicated functional independence.	Calculation of IADL scores for evaluation of functional independence in activities of daily living.
**Comorbid conditions**	Self-Administered Comorbidity Measure (SCM)	The SCM is a self-reported questionnaire consisting of items assessing various medical conditions, such as hypertension, diabetes, heart disease, and cancer. Participants will indicate their diagnosis and if the diagnosis interferes with their ability to carry out daily life activities.	Identification of comorbid diagnoses and assessment of overall health status.
**Cognitive Functioning**	Montreal Cognitive Assessment (MoCA), developed by Ref. 105	The MoCA is a standardized cognitive screening tool comprising various tasks assessing different cognitive domains, including memory, attention, language, and visuospatial abilities. Trained staff will administer the MoCA to participants in a quiet and distraction-free environment. Scores will be calculated based on participants' responses, with scores ≤26 indicating possible cognitive impairment.	Calculation of MoCA cognitive function scores (score range: 0-30).
**Mood**	Geriatric depression scale (GDS) short form, developed by Ref. 106	The GDS short form is a validated questionnaire designed to evaluate mood among older adults. The scale assesses depressive symptoms in older adults, encompassing various aspects of depression, including mood, motivation, energy level, social engagement, and cognitive functioning. The GDS short form comprises 15 items and scores will be calculated based on participants' responses, with scores of >5 indicating depressive symptoms.	Calculation of GDS scores (score range: 0-15).
**Ocular Health**	Self-report form	Participants are given a form to self-report any vision-related issues.	Identification of vision-related symptoms, eye conditions, and overall self-report ocular health status.

**Telephone interview**

*Aim*

The preliminary telephone-based interview aims to understand older adults’ current practices and preferences related to health, light exposure, and technology. We assess participants’ baseline technological and health literacy levels during the telephone interview stage to determine support requirements in conducting discussions with them.

*Procedure*

Participants will receive a scheduled appointment for the telephone interview, communicated well in advance with a reminder closer to the appointed date and time. During the scheduled call time, participants will receive an introductory briefing over the phone, providing an overview of the study’s objectives to ensure a comprehensive understanding of the research aims. The telephone interview will proceed with administering questionnaires evaluating physical activity levels, and sleep quality. Additionally, the interview will include questions exploring participants’ practices and preferences regarding light exposure, smartphone use, and mobile health literacy. Participants’ responses will be carefully recorded to accurately capture all relevant information. The telephone interview is anticipated to last approximately 30 minutes.

*Measurements & outcomes*

The telephone interviews will serve as a platform for administering questionnaires on physical activity and sleep, as well as structured interview questions on light exposure and mHealth. By analysing the responses, we aim to gain insights into their status and identify common trends and patterns among the participants, thereby enhancing our understanding of their attitudes and behaviours before exposure to the LightSPAN mHealth intervention. This comprehensive overview of participant characteristics will inform subsequent stages of the study and guide the development of targeted interventions tailored to the needs and preferences of older adults. Table 4 and Table 5 outline the constructs that will be measured during the telephone interview using standardised questionnaires and structured interview questions.

**Table 4.** Overview of telephone interview measurements and outcomes utilizing standardized questionnaires for assessing physical activity and sleep quality in older adults.

Construct	Instrument	Administration and instrument details	Outcome
**Physical activity**	Physical Activity Scale for the Elderly (PASE) ^107^	The PASE assesses self-reported physical activity levels in older adults. The scale is a widely used tool for evaluating the frequency, duration, and intensity of various types of physical activities commonly performed by older individuals. PASE assesses multiple dimensions of physical activity, including leisure, household, and occupational activities. The questionnaire consists of 26 items, and each item is assigned a weighted score based on the frequency and duration of the activity, with higher scores indicating higher levels of physical activity. The total score is calculated by summing the weighted scores for all items, providing an overall measure of the individual's physical activity level.	Calculation of PASE score for physical activity levels in older adults
**Sleep**	Pittsburgh Sleep Quality Index (PSQI) ^108^	The PSQ is a self-report questionnaire evaluating various facets of sleep quality in adults over the previous month, encompassing subjective sleep perception, latency to sleep onset, duration of sleep, efficiency of habitual sleep, frequency of disturbances during sleep, use of sleep medications, and the impact of sleep problems on daytime functioning. Comprising 19 items, each item is scored on a scale from 0 to 3, and the scores across domains are summed to generate a global score ranging from 0 to 21. Higher scores indicate poorer sleep quality, with a global score of 5 or above often indicating significant sleep disturbances.	Calculation of PSQI scores for assessment of sleep quality and disturbances

**Table 5.** Overview of telephone interview measurements and outcomes using structured interview questions for assessing light exposure, health literacy, smartphone use, and mobile health literacy in older adults.

Construct	Instrument	Administration details	Outcome
**Light exposure**	Structured interview questions	Administered during telephone interview	Participants’ light exposure preferences and behaviours
**Health Literacy**			Participants’ perceived health literacy
**Smartphone Use**			Participants’ smartphone usage patterns, activities, and preferences
**Mobile Health Literacy**			Participants’ familiarity with mobile health apps

**Focus group discussion**

*Aim*

The FGD aims to explore older adults’ perspectives, needs, and preferences regarding light exposure, motivations for behaviour change, and acceptance of mHealth interventions. Also, the LightSPAN mHealth intervention will be introduced, and feedback on the proposed intervention plan will be gathered. This exploration aims to establish a foundational understanding of their perspectives, define and describe potential challenges and opportunities for effective intervention uptake, and identify areas for improvement to support the intervention’s effectiveness and acceptance among the target users.

*Procedure*

The session will commence with introductions and an icebreaker activity to foster a comfortable atmosphere conducive to open discussion. Participants will then be provided an overview of the FGD’s objectives to establish context and clarify expectations. Guided by the moderator, discussions will encompass various topics, including participants’ experiences and perceptions of light exposure, motivations for behaviour change, smartphone usage patterns, and acceptance of mHealth interventions. Participants will then be given the background of the LightSPAN project and the LightSPAN mHealth intervention plan, for which their feedback and initial acceptability will be sought. Subsequently, a guided questionnaire will be administered to gather preferences on various app components. The FGD will conclude with a wrap-up session summarising key points and outlining the next steps. The session will last 90 minutes, encompassing introductions, instructions, discussion, and wrap-up. Sessions will include up to five participants.

*Measurements & outcomes*

The FGDs will be guided by a developed script containing main questions and subsidiary prompts. The sessions will be audio recorded using a recording device and supplemented with notetaking to ensure thorough documentation of participants’ responses and interactions. The session covers three main components: user perceptions and attitudes, feedback on the LightSPAN mHealth intervention plan, and the feature preference questionnaire, detailed in the table below. Their responses will be analysed to identify recurring themes, patterns, and commonalities, thereby providing insights into issues, concerns, and preferences pertinent to light exposure management and mHealth interventions. These insights will serve as valuable inputs to inform further intervention development and app design iterations, facilitating the creation of a more effective and user-centric solution. Table 6 below presents the constructs that will be explored, the instruments that will be used, the administration details, and the specific outcomes that will be assessed during the FGD.

**Table 6.** Overview of focus group discussions measurements and outcomes on user experiences, attitudes, and feedback towards the LightSPAN mHealth intervention.

Construct	Instrument	Administration details	Outcomes
**User experiences and attitudes**			
**Light exposure**	Open-ended questions	Participants will be engaged through a series of tailored questions to delve each construct	• Awareness of light exposure and its impact • Daily practices • Barriers and facilitators
**Motivations for behaviour change**	Open-ended questions		• Factors influencing and inhibiting motivation to adopt healthy behaviour
**Smartphone usage**	Open-ended questions		• Comfort levels with smartphone usage. • Perception of smartphone importance in daily life • Frequency of smartphone usage and typical activities • Perception of smartphone impact on daily life • Perceived ease or difficulty of smartphone usage
**Acceptance of mHealth Interventions**	Questions are adapted from the m-health acceptance model ^109^ integrating elements of Self-Determination Theory (SDT), Task-Technology Fit (TTF), and the Technology Acceptance Model (TAM)		• Participants' initial reactions to using a health monitoring smartphone app. • SDT: Feelings of autonomy, relatedness, and competence when using mHealth apps • TTF: Alignment between mHealth and participants' health management practices and needs. • TAM: Perceived usefulness, and ease of use, and usage behaviours of mHealth services (frequency and patterns of using mobile health services for managing health)
**Feedback of the LightSPAN mHealth Intervention**			
**Implementation of LightSPAN intervention**	Presentation of LightSPAN mHealth intervention plan and open-ended questions	Participants are provided with background information then asked to provide feedback on the proposed intervention	• Initial thoughts on the intervention plan • Perception of incorporating a light sensor and wrist-worn fitness tracker into daily routine
**Acceptance of intervention design factors**	Questions are crafted based on seven dimensions consisting interest, intent benefit, enjoyment, utility, confidence and difficulty, adapted from Ref. 110 which is based on constructs from the Theory of Planned Behaviour as well as prior assessments of behavioural motivation and mHealth design preferences	Participants are asked questions according to the dimensions to determine their initial perceptions of acceptability towards the LightSPAN mHealth intervention	• Interest in participating. • Likelihood of participation • Perceived benefits of participating and beliefs in its effectiveness for achieving good light exposure. • Anticipation of enjoyment from participating in the intervention • Perceived utility of the app in addressing participants' needs and challenges related to light exposure. • Level of confidence in using the app and engaging with it • Perceived difficulty of participating in the intervention and anticipated challenges or obstacles
**Preferences and desired features**	Self-developed feature preference questionnaire (FPQ) based on the LightSPAN mHealth app	Guided survey administered at the end of the FGD session	• Calculation of FPQ responses across 4 components; app features, interactive components for app engagement, app usage and app support consisting of 34 items , each rated on a scale of importance and interest

**Prototyping workshop**

*Aim*

The prototyping workshop aims to present the prototype of the LightSPAN mHealth app and collect feedback for its iterative development and refinement.

*Procedure*

The prototyping workshop will be interactive, showcasing the prototypes of the LightSPAN mHealth app to the participants. The prototype includes the essential features and functionality for initial testing and user feedback. At the start of the workshop, participants will be briefed on the workshop’s goals and roles. The prototypes will be presented in a storyboard format, broken down into key user flows or scenarios. Each storyboard illustrates user actions, system responses, and interface elements. Participants will be encouraged to share their initial thoughts and provide detailed feedback, highlighting aspects they liked and disliked, assessing user flow navigation, and suggesting improvements or changes. Subsequently, participants will engage in a design preference voting activity, where they will be presented with mock-ups of design options. They will cast their votes for their preferred option, and the votes will be recorded. After collecting the votes, participants will be encouraged to share the reasoning behind their choices for deeper insights into their preferences. The workshop will conclude with a summary of key insights gathered and outline the next steps in development. The session will be allocated 90 minutes, including introductions, prototype activity, and session wrap-up.

*Measurements & outcomes*

The prototyping workshop seeks to gain insights from participants’ feedback, validate design choices, and identify areas for improvement. This process will involve pinpointing areas of strength that resonate positively with the participants and highlighting areas requiring refinement based on concerns or issues raised during the workshop. Additionally, the process will include identifying commonalities, trends, and areas of consensus or divergence among participants regarding the prototype designs.

Data will be documented primarily through participant verbatim comments and feedback on prototypes (audio data), votes, and researcher observations. The discussion guide, outlining key topics and questions, will structure the workshop, focusing on two main areas: user perceptions and feedback and design preferences. Table 7 provides a synthesis of the constructs that will be explored, along with the instruments that will be used, the administration details, and the intended outcomes during the prototyping workshop.

**Table 7.** Overview of prototyping workshop measurements and outcomes for LightSPAN mHealth app development.

Construct	Instrument	Administration details	Outcomes
**User perceptions and feedback**	Prototypes of LightSPAN mHealth app and open-ended questions	Presentation of prototype in storyboarding format followed by discussion soliciting participants’ feedback and input	• Initial impressions • Identification of likes and dislikes. • Perception of app's navigational flow • Identification of potential challenges and suggestions for refinement
**Design preferences**	Slide deck presentation displaying design options and open-ended questions	Voting-style activity followed by discussion exploring participants reasoning	• Preference for design options indicated by participant votes. • Insight into factors influencing participant design choices

### Data analysis plan

The qualitative and quantitative findings will be integrated to provide a holistic understanding of the co-design process and its outcomes.

### Descriptive analysis

Quantitative data analysis will be conducted using R (version 2023-10-31), R Studio (version 2023.12.1+402), ^111^ Python (version 3.12.1) or future versions ^112^ if available at the time of analysis. This will enable the application of statistical techniques to analyse numerical data collected during the study, primarily descriptive statistics. Standard descriptive statistics, e.g., mean, standard deviation, minimum, and maximum, will be reported for all metric variables. Frequencies and percentages will be reported for categorical variables.

### Thematic analysis

Qualitative data will be analysed using thematic analysis, a widely used method for identifying, analysing, and reporting patterns and themes within data. Participant verbatim discussions (audio data) will be transcribed and organised alongside detailed notes taken during the sessions to capture nuances and key insights. The analysis will be supported by software such as NVivo (Lumivero, United States), which aids in organising and managing qualitative data. Two independent data coders will be involved in the coding process, ensuring reliability and consistency in identifying themes and patterns.

### Outputs

The current study will yield a co-designed app delivered intervention program tailored to the needs of older adults, which will then be carried onto two additional trials: a pilot and feasibility trial and a randomised controlled trial (RCT) for further evaluation of the intervention’s ability to modify light exposure, and sleep and other health-related outcomes. The development process entails two components, each documented for academic dissemination. The first component is data obtained through the co-design process with older adults. A research paper will report this process, presenting the findings and analysis of their perspectives, preferences, and experiences. Concurrently, the second component is the development of the LightSPAN mHealth app. This process will also be documented to provide a detailed account of the technical aspects of the creation, including the utilisation of user-centric design principles and the underlying source code.

## Discussion

As the global population ages, formulating and tailoring health interventions to meet the needs of older adults becomes increasingly vital. The LightSPAN project aims to highlight the importance of optimising light exposure in daily life between ageing, light exposure, and health outcomes, particularly among community-dwelling older adults in Singapore. Through a collaborative and iterative co-design process, we have outlined our framework to co-create an intervention attuned to this demographic’s unique needs and preferences. Intervening at the intersection of ageing and light exposure, the LightSPAN project, anchored in behaviour change techniques and mobile technology, seeks to empower older adults to actively manage their light exposure behaviours to promote overall well-being. However, in doing so, it is important to formulate and tailor the health intervention to meet the needs of older individuals.

The present co-design protocol delineates the formative steps of the LightSPAN project, presenting the systematic procedures undertaken to develop a mHealth behaviour change intervention to optimise light exposure among Singapore’s older adults. Engaging with older adults throughout the intervention design process is crucial to ensure their perspectives, needs, preferences, and user experiences are effectively accounted for. By considering both the characteristics of participants and the design elements of the mHealth interventions, we aim to carry out an intervention that maximises user engagement, adherence, and overall impact on health outcomes. Nonetheless, despite the promising potential of the LightSPAN project, engaging older adults in the co-design process may present inherent challenges, as reported in previous literature. These challenges include recruitment and retention difficulties, logistical and scheduling complexities, competing personal commitments, group dynamics, varying levels of motivation to participate, and disparities in literacy levels. ^113, 114^

To address these challenges, we have proactively incorporated several strategies into our protocol. ^60, 113^ These include partnering with community organisations to assist with recruitment, setting a date for the sessions several weeks in advance to accommodate participants’ schedules, providing participants with educational materials to enhance their understanding of the project, offering context and a clear overview of aims at the beginning of each session, clarifying roles, and incorporating mobility breaks to maintain engagement and prevent fatigue. Additionally, we will conduct guided surveys to assist older adults in completing questionnaires and ensure comprehension and accuracy, and probe for literacy in telephone interviews. Furthermore, we aim to communicate using simple language and utilise visual aids to facilitate understanding while also scheduling sessions in the morning to coincide with participants’ peak alertness.

Additionally, we will maintain small group sizes to foster a conducive environment for participation. Moreover, we recognise the importance of building rapport and trust with participants and will pay special attention to establishing a good working relationship. From the researcher team’s end, debrief sessions will be conducted to review each session and contour the next steps in the development process accordingly.

To complete the co-design process and ensure the comprehensive development of the LightSPAN mHealth intervention, we will pilot-test the intervention as part of the final and third workstream. A pilot study is planned to evaluate the feasibility of the LightSPAN intervention before broader implementation. This pilot study will provide valuable insights into the practicalities of intervention delivery, user engagement, and potential areas for refinement. Subsequently, a post-pilot discussion will be conducted to explore participants’ perceptions and experiences and assess the usability and acceptability of the intervention to further refine the intervention. This iterative approach to intervention development ensures that the LightSPAN intervention remains responsive to the needs and preferences of older adult users.

## Conclusion

In conclusion, the LightSPAN project presents a promising approach to optimising light exposure among older adults in Singapore. This protocol outlines a systematic approach for developing an mHealth behaviour change intervention tailored to optimise light exposure among older adults in Singapore. Through engagement with older adults and key stakeholders, this protocol addresses various aspects of light exposure optimisation, incorporating insights from literature reviews, consultations, telephone interviews, focus group discussions, and interactive prototyping workshops. By actively involving community service providers and older adults in the co-design process, we aim to create an intervention that is not only evidence-based but also user-centred and culturally appropriate. The LightSPAN project seeks to improve the well-being and quality of life of older adults and contribute valuable insights to the field of mHealth interventions for healthy ageing.

### Ethical considerations

Ethical approval has been obtained by the Parkway Independent Ethics Committee (PIEC) under approval number PIEC/2024/030, dated 2 October 2024. All procedures will adhere to the ethical principles outlined in the Declaration of Helsinki, including obtaining written informed consent from participants, ensuring confidentiality, and protecting participants’ rights, privacy and well-being.

## Author contributions

*Conceptualisation*: ZAA, RRM, MS

*Formal Analysis*: ZAA, RRM

*Funding acquisition*: MS

*Methodology*: ZAA, RRM, MS, JW

*Project administration*: RRM, MS

*Software*: DDV

*Supervision*: MS

*Visualisation*: ZAA, RRM

*Writing – original draft*: ZAA

*Writing – review & editing*: ZAA, RRM, DDV, JW, MS

## Data Availability

No data are associated with this article. This protocol follows the Consolidated Criteria for Reporting Qualitative Research (COREQ) guidelines. ^115^ Data are available under the terms of the Creative Commons Attribution 4.0 International license (CC-BY 4.0).

## Software availability

All code used in the project will be made publicly available. Analyses will be performed using R, an open-source and freely accessible software. For qualitative data analysis, we will use NVivo, a proprietary tool. However, a similar analysis can also be conducted using Taguette (a free, open-source alternative) or performed manually by organizing and coding data in spreadsheets or word processors.
