# Supplementary material for: Protocol for a co-design study for the development of a chronotherapeutic mobile health behaviour change intervention targeting light exposure among older adults
Source: F1000Res. 2025 Jul 25;13:1356. Originally published 2024 Nov 12. [Version 2] doi: 10.12688/f1000research.157814.2 (PMC12361882; doi:10.12688/f1000research.157814.2)
Supplement: Peer review history

# Reviewer report: Reviewer response for version 2

**DOI:** 10.5256/f1000research.183669.r400457

**Published:** 2025-11-05

**Authors:** Nicholas Meyer

**Affiliations:** 1 King's College London, London, UK; 2 Insomnia and Behavioural Sleep Medicine Clinic, University College London Hospitals NHS Foundation Trust, University College London, London, UK

Thank you for your responses and amendments, which fully answer my points. 

 No further comments.

Is the study design appropriate for the research question?

Yes

Is the rationale for, and objectives of, the study clearly described?

Yes

Are sufficient details of the methods provided to allow replication by others?

Yes

Are the datasets clearly presented in a useable and accessible format?

Not applicable

Reviewer Expertise:

Sleep and circadian rhythms; mealth.

I confirm that I have read this submission and believe that I have an appropriate level of expertise to confirm that it is of an acceptable scientific standard.

# Reviewer report: Reviewer response for version 2

**DOI:** 10.5256/f1000research.183669.r400459

**Published:** 2025-08-18

**Authors:** Kiran Maini Gerhardsson

**Affiliations:** 1 Lund University, Lund, Sweden

Thank you for clarifying and adding the necessary details regarding the sample and study flow. However, please check the placement of figures:

 P 6: Figure 1 does not display the study flow diagram.

 P 9: Figure 2 does not display the application of the SDT.

Is the study design appropriate for the research question?

Yes

Is the rationale for, and objectives of, the study clearly described?

Yes

Are sufficient details of the methods provided to allow replication by others?

Partly

Are the datasets clearly presented in a useable and accessible format?

Not applicable

Reviewer Expertise:

Environmental psychology, applied gerontology, and lgiht and health

I confirm that I have read this submission and believe that I have an appropriate level of expertise to confirm that it is of an acceptable scientific standard.

# Reviewer report: Reviewer response for version 1

**DOI:** 10.5256/f1000research.173318.r344591

**Published:** 2025-01-28

**Authors:** Nicholas Meyer

**Affiliations:** 1 King's College London, London, UK; 2 Insomnia and Behavioural Sleep Medicine Clinic, University College London Hospitals NHS Foundation Trust, University College London, London, UK

The focus of the current protocol is around the co-design of an mHealth behaviour change intervention targeting light exposure in older adults. The authors have put a great deal of thought into the steps through which an acceptable intervention can be developed, incorporating co-production approaches and mHealth implementation theory. All of this seems excellent and I have no further comments on these aspects.

 An mHealth intervention that is beautifully designed in terms of acceptability, usability and adherence, would nonetheless be of little utility if it didn’t lead to improvements in health outcomes. It is not so clear from this proposal which health outcomes the intervention is seeking to improve, other than those that will be identified in the literature review in workstream 1a. I therefore wonder if it would be helpful, at this early stage, if the authors put greater *a priori *consideration into the what problems the proposed intervention are seeking to solve, in what populations, and how this will ultimately be tested? Much of this proposal appropriately seeks to develop the intervention in a ‘bottom up’ manner, with end-user input, but first setting the broad parameters of the intervention in a ‘top-down’ fashion also seems important.

 For example, it seems the intervention will be designed for non-clinical populations - i.e. healthy older adults without significant cognitive decline or psychiatric problems. Is the final light intervention intended to be used as a preventative tool for reducing risk of transition to clinical disorders such as mild cognitive impairment, dementia or mood disorder? Or will the goal of the intervention be to improve daily functioning such as positive mood, energy levels, physical activity, in non-clinical groups? These are important considerations, as it will help the participants in the co-design study to be better able to frame their recommendations.

 This may be the topic of a separate protocol, however it may also be helpful to consider how the final intervention will be evaluated - in a cohort of healthy older adults, who will be followed up in a randomised-controlled study? Or at a population-level study, looking at broader health and wellbeing outcomes on a larger scale? Which outcome measures will be examined? Again, this speaks to whether the intervention is envisioned to be a tool for clinical populations and targeted disease prevention, or a wider population level intervention. 

 I appreciate much of these points are implicit in the protocol, however making them more explicit may benefit the broader aims of the study.

Is the study design appropriate for the research question?

Yes

Is the rationale for, and objectives of, the study clearly described?

Yes

Are sufficient details of the methods provided to allow replication by others?

Yes

Are the datasets clearly presented in a useable and accessible format?

Not applicable

Reviewer Expertise:

Sleep and circadian rhythms; mealth.

I confirm that I have read this submission and believe that I have an appropriate level of expertise to confirm that it is of an acceptable scientific standard, however I have significant reservations, as outlined above.

# Response

**Published:** 2025-06-13

**Authors:** Manuel Spitschan

**Affiliations:** Not Applicable, Germany

**R3.1**

**The focus of the current protocol is around the co-design of an mHealth behaviour change intervention targeting light exposure in older adults. The authors have put a great deal of thought into the steps through which an acceptable intervention can be developed, incorporating co-production approaches and mHealth implementation theory. All of this seems excellent and I have no further commentss on these aspects.**

 We thank the reviewer for their positive comments.

**R3.2**

**An mHealth intervention that is beautifully designed in terms of acceptability, usability and adherence, would nonetheless be of little utility if it didn’t lead to improvements in health outcomes. It is not so clear from this proposal which health outcomes the intervention is seeking to improve, other than those that will be identified in the literature review in workstream 1a. I therefore wonder if it would be helpful, at this early stage, if the authors put greater a priori consideration into the what problems the proposed intervention are seeking to solve, in what populations, and how this will ultimately be tested? Much of this proposal appropriately seeks to develop the intervention in a ‘bottom up’ manner, with end-user input, but first setting the broad parameters of the intervention in a ‘top-down’ fashion also seems important.**

 We fully agree that an effective mHealth intervention must ultimately demonstrate impact on relevant health outcomes. While the co-design approach emphasizes bottom-up input from end users, our intervention is also grounded in a top-down framework. The proposed LightSPAN intervention is designed to support older adults by improving outcomes related to circadian regulation, sleep quality, mood, alertness, and cognitive function, and is guided by recommendations from Brown et al. (2022), which outline targets for minimum/maximum melanopic EDI across the daytime, pre-sleep, and night-time periods. We acknowledge the importance of clearly defining the problems the intervention seeks to address and will expand on these target domains in the Randomised Controlled Trial (RCT) intervention protocol.

**R3.3**

**For example, it seems the intervention will be designed for non-clinical populations - i.e. healthy older adults without significant cognitive decline or psychiatric problems. Is the final light intervention intended to be used as a preventative tool for reducing risk of transition to clinical disorders such as mild cognitive impairment, dementia or mood disorder? Or will the goal of the intervention be to improve daily functioning such as positive mood, energy levels, physical activity, in non-clinical groups? These are important considerations, as it will help the participants in the co-design study to be better able to frame their recommendations.**

 The current intervention is designed for non-clinical populations, specifically, generally healthy older adults. Future work may explore its adaptation and applicability for more vulnerable or clinically relevant populations.

**R3.4**

**This may be the topic of a separate protocol, however it may also be helpful to consider how the final intervention will be evaluated - in a cohort of healthy older adults, who will be followed up in a randomised-controlled study? Or at a population-level study, looking at broader health and wellbeing outcomes on a larger scale? Which outcome measures will be examined? Again, this speaks to whether the intervention is envisioned to be a tool for clinical populations and targeted disease prevention, or a wider population level intervention.**

 We are currently developing the protocol for pilot and feasibility trial, and the main RCT. This will be the subject of a separate protocol publication.

**R3.5**

**I appreciate much of these points are implicit in the protocol, however making them more explicit may benefit the broader aims of the study.**

 We appreciate the reviewer’s points and have made several aspects more explicit in our manuscript.

# Reviewer report: Reviewer response for version 1

**DOI:** 10.5256/f1000research.173318.r344589

**Published:** 2025-01-03

**Authors:** Kathryn Reid

**Affiliations:** 1 Feinberg School of Medicine, Northwestern University, Chicago, IL, USA

Increasing evidence supports the role of light exposure patterns as a contributing factor to health. The protocol to develop the mHealth LightSPAN app as described has the potential to have a significant impact on the health of older adults. Overall, the rationale and approach are clear, although it would benefit from some additional details.

 It would be useful to provide some examples of the type of approaches that the research team will build into the mHealth application. The authors mention that there are evidence-based recommendations for light exposure patterns but that there are no “practical recommendations” of how to achieve these. While it is clear that the proposed research aims to provide these practical recommendations, some potential examples would be beneficial to the reader. 

 On page 4 there is mention of the project “developing and testing an mHealth behaviour change intervention”, as described there is no testing of the application in the current proposal. 

 In addition, in the same paragraph (page 4) it sounds as if the application already exists see ”LightSPAN mHealth app, which tracks light exposure using a light sensor and employs various behaviour-change strategies to optimise light exposure patterns”. This makes it unclear whether the goal is to develop a new application or to optimize an existing application for older adults?

 Work stream 1a-1d as reported are clear and well structured.

 Very little information is provided for Workstream 2a, for example there is no description of how the community service providers will be selected, recruited and what characteristics they will have. For example, will they be physicians (what specialty general medicine, neurology etc.) nurses, and researchers will they be hospital based or from community clinics or a mix? Will they be exclusively from Singapore? What will be the number of providers interviewed. There are also no specifics provided on whether these interviews will be structured and what information will be collected and how this will be used to develop the LightSPAN mHealth application.

 Work stream 2b study sample selection, will prior use of mHealth or light treatment be an exclusion factor?

 There will be 20 older adults selected for participation. It would be useful to provide some information about whether the selection of participants will be balanced for any demographic characteristics, such as age, sex, socioeconomic status, education etc. 

 Another consideration is where will the participants be recruited from, specifically will they be from one or several of the Active Ageing Centre’s to enhance diversity or perspectives.

 There is brief mention of the sample of 20 being split into 4 groups and individuals, how will this be achieved, is it random or based on location? 

 Will the prototyping workshop include the same 20 participants as for telephone interview and focus groups?

 How will attrition be handled if there is dropout between the telephone interview, focus groups and prototype workshop? What is the minimum number of participants to be included in each of these phases.

 What is the proposed timeline for the different phases of the study?

 The authors propose moving from the proposed protocol phase of co-designing the mHealth application into a randomized control trial. An initial pilot trial for assessment of feasibility and acceptability would be advised. Also what would the outcomes of the proposed trial be, would it simply be optimized light exposure patterns?

Is the study design appropriate for the research question?

Yes

Is the rationale for, and objectives of, the study clearly described?

Yes

Are sufficient details of the methods provided to allow replication by others?

Partly

Are the datasets clearly presented in a useable and accessible format?

Not applicable

Reviewer Expertise:

Sleep and Circadian Science, Aging

I confirm that I have read this submission and believe that I have an appropriate level of expertise to confirm that it is of an acceptable scientific standard, however I have significant reservations, as outlined above.

# Response

**Published:** 2025-06-13

**Authors:** Manuel Spitschan

**Affiliations:** Not Applicable, Germany

**R2.1**

**Increasing evidence supports the role of light exposure patterns as a contributing factor to health. The protocol to develop the mHealth LightSPAN app as described has the potential to have a significant impact on the health of older adults. Overall, the rationale and approach are clear, although it would benefit from some additional details.**

 We thank the reviewer for their careful review.

**R2.2**

**It would be useful to provide some examples of the type of approaches that the research team will build into the mHealth application. The authors mention that there are evidence-based recommendations for light exposure patterns but that there are no “practical recommendations” of how to achieve these. While it is clear that the proposed research aims to provide these practical recommendations, some potential examples would be beneficial to the reader.**

 We intend to incorporate a range of behaviour change techniques and tailored guidance to support optimal light exposure throughout the day. For example, the mHealth app may include:

- Daytime prompts to encourage outdoor activity or sitting near a window
- Evening tips to reduce light exposure before bed (e.g., dimming lights)
- Activity suggestions to integrate light exposure into routines (e.g., reading or exercising by a window);
- Feedback visualisations to help users track their light exposure patterns and progress

These features will be supported by educational modules, reminders, and sensor-based light data.

**R2.3**

**On page 4 there is mention of the project “developing and testing an mHealth behaviour change intervention”, as described there is no testing of the application in the current proposal.**

 We have now clarified the text, and state: “This protocol concerns the co-design stage of the LightSPAN project, which will be followed by a pilot and feasibility trial, and a randomized controlled trial (RCT).”

**R2.4**

**In addition, in the same paragraph (page 4) it sounds as if the application already exists see ”LightSPAN mHealth app, which tracks light exposure using a light sensor and employs various behaviour-change strategies to optimise light exposure patterns”. This makes it unclear whether the goal is to develop a new application or to optimize an existing application for older adults?**

 Thank you for highlighting this point. We have revised in the paragraph to clarify that the LightSPAN mHealth app is a novel app.

**R2.5**

**Work stream 1a-1d as reported are clear and well structured. Very little information is provided for Workstream 2a, for example there is no description of how the community service providers will be selected, recruited and what characteristics they will have. For example, will they be physicians (what specialty general medicine, neurology etc.) nurses, and researchers will they be hospital based or from community clinics or a mix? Will they be exclusively from Singapore? What will be the number of providers interviewed. There are also no specifics provided on whether these interviews will be structured and what information will be collected and how this will be used to develop the LightSPAN mHealth application.**

 We have revised the text to clarify the recruitment process, participant characteristics, and data collection approach for Workstream 2a. The consultation will take the form of a structured focus group discussion, guided by a topic outline.

**R2.6**

**Work stream 2b study sample selection, will prior use of mHealth or light treatment be an exclusion factor?**

 Co-design: No exclusion based on participation in other trials, but we will do this in RCT.

**R2.7**

**There will be 20 older adults selected for participation. It would be useful to provide some information about whether the selection of participants will be balanced for any demographic characteristics, such as age, sex, socioeconomic status, education etc.**

 Participant recruitment will not be stratified or balanced based on specific demographic characteristics such as socioeconomic status or education level. However, we will aim for diversity in age and sex during group formation to encourage a range of perspectives during discussions. Demographic data will also be collected and reported to provide context for interpreting the co-design findings.

**R2.8**

**Another consideration is where will the participants be recruited from, specifically will they be from one or several of the Active Ageing Centre’s to enhance diversity or perspectives.**

 We have clarified that we will recruit from two AACs.

**R2.9**

**There is brief mention of the sample of 20 being split into 4 groups and individuals, how will this be achieved, is it random or based on location?**

 We have now clarified this to be: “This sample will be divided into four groups comprising five participants matched in sex and age”

**R2.10**

**Will the prototyping workshop include the same 20 participants as for telephone interview and focus groups?**

 Yes.

**R2.11**

**How will attrition be handled if there is dropout between the telephone interview, focus groups and prototype workshop? What is the minimum number of participants to be included in each of these phases.**

 At least 20, and we have left buffer for recruitment. If the number i sunder 20, we will change recruitment strategy and replace from community. We accept attrition as part of any study and will report as part of the process.

**R2.12**

**What is the proposed timeline for the different phases of the study?**

 For workstream 2, each phase will be scheduled approximately one month apart to allow time for data analysis, iterative planning, and preparation between phases. The overall timeline for data collection is expected to span approximately 4 months.

**R2.13**

**The authors propose moving from the proposed protocol phase of co-designing the mHealth application into a randomized control trial. An initial pilot trial for assessment of feasibility and acceptability would be advised. Also what would the outcomes of the proposed trial be, would it simply be optimized light exposure patterns?**

 We are planning to run a pilot and feasibility trial, and now write this in the manuscript. We will examine light exposure, and sleep and other related outcomes in this pilot and feasibility trial.

# Reviewer report: Reviewer response for version 1

**DOI:** 10.5256/f1000research.173318.r344595

**Published:** 2024-12-26

**Authors:** Kiran Maini Gerhardsson

**Affiliations:** 1 Lund University, Lund, Sweden

Review report

 Drawing on the title (co-design), I expected more active collaboration with stakeholders and target users throughout the study – from start to end. I would characterise the study as a two-part (or two-phase) development of a health-promoting intervention. The intervention targets light exposure among older adults. The first phase (referred to as Workstream 1 by the authors) consists of foundational activities resulting in an early prototype of a mobile phone application, which is followed by a second stage (Workstream 2), including continued development and evaluation by future community service providers and target users (community-dwelling older adults, age 60 and over). Thus, a co-design approach will be applied in the second phase. The intervention – delivered via a mobile app – will be refined between the two phases and within the second. 

 I do not suggest any changes to the protocol but rather an explication of what the authors mean by a co-design approach (to justify the title), minor elaborations on some procedure details and that the Background section is shortened to make space for an extended paragraph on co-design, situating the current study in more detail in the area of co-design, (e.g., addressing the frequency of co-designers’ involvement, timing – where in the process – and influence on the design solutions).

 I look forward to reading the results when the study is completed. Light-related interventions directed at older adults are much needed.

 Below are some questions asking for clarification or suggestions, hoping they will improve the manuscript before indexing.

 Abstract: “open-ended discussions”. Could you be more specific – workshops, group interviews, focus group discussions?

 Abstract (the final sentence): “... will ensure that the intervention addresses the needs of its future users.” Consider rephrasing to, for example: “facilitate future implementation”. You will probably not capture the needs of all future users (considering the rapid development of technology). However, a limited number of participants will certainly help to capture many potential issues.

 P. 3: (subtitle) “ageing health” –> “healthy ageing”. Also, this subsection could be shorter. A background section should not be overly long in a study protocol.

 P. 3: “Exposure to bright light, particularly natural sunlight, ...” Sunlight or daylight?

 P. 3: “To address this, ...” Challenges, issues?

 P. 4: “Overall, studies co-designing technologies for health management, fitness, or in-home wellness have recognised the importance of including older adults as co-designers.” Missing word: “studies of”?

 P. 4: “ensuring maximum feasibility and sustainability.” Word choice – maybe more cautious – “facilitating” or “promoting”. Considering the rapid technology development and often long co-design processes, it might be better to be a bit cautious.

 P. 4: Word choice: “urging them”. Maybe “supporting” or “encouraging them”?

 P. 4: “The intervention utilises the LightSPAN mHealth app...” Confusing – is the app already developed? Or should it be rephrased to “will be provided using the ...”

 P. 4: “... the potential efficacy of a technology-driven, lifestyle-oriented intervention ...”. There is a misalignment here in giving technology priority. Your intention is to develop a user-centred intervention to address lifestyle issues (“... the potential efficacy of a lifestyle-oriented intervention using smartphone technology...”).

 P. 5: “mHealth user personas, ...”. Using personas is a helpful initial tool in the early step of development until you have conducted the interviews with the target users. Have you also considered using the interview findings in the next step (workstream 2) to create project-specific personas? Personas based on your interviews might be more helpful for further developing the intervention than more generic user profiles.

 P. 5 and 6: The workstreams described on page 5 do not entirely reflect Figure 1 on page 6. For example, is the prototype developed before workstream 2 (as shown in Figure 1, "initial development of LightSPAN mHealth app), or is it included in workstream 2 as the workstream title indicates on page 5 ("Co-design og the mHealth intervention")? 

 Also, I find new terms in Figure 1 a bit confusing, such as "LightSPAN Behaviour Change Model" and "infographic with recommendations for mHealth design". 

 In Figure 1, a LightSPAN mHealth prototype app is developed after engaging with community service providers but before engaging with older adults. 

 The unclarities mentioned above make me wonder whether this is a co-design project or a user-centred development of intervention, including need assessments and user evaluations.

 Relating to this, in the conclusion on page 5, the authors write: "A co-design approach will be employed to develop an effective mHealth intervention tailored to our target users, involving active collaboration between the researchers and older adult participants." But Figure 1 shows something slightly different: you will develop an early prototype – get feedback from the community service provider – refine the prototype - and then you will ask older adults about their needs and preferences – and finally get feedback on the prototype by older adults participating in focus groups. However, the procedure described in the study might be regarded as a co-design study in a weaker sense. Then, the authors could describe their definition of a co-design approach.

 P. 6: Co-design team. The co-design team, as named by the authors, includes researchers and a coordinator, but no stakeholders or target users as expected. Why not refer to the team as a research team?

 P. 6: "The team will be assigned roles ..." Do you mean during the workshop and focus groups? Will all team members participate? What is the difference between a moderator and a facilitator?

 P. 6: Guiding principles. The principles are well described and essential to the intervention's development. To achieve the design objectives, the authors highlight, for example, "To facilitate this, tailored interventions must be delivered appropriately for older adults, ..."

 I wonder if you have considered how you will manage input from the stakeholders or end users concerning intervention delivery. An app-based solution may not be ideal. What if they express preferences for in-person meetings? Or getting tech support (on more occasions than at the introductory session)? Or reading the intervention content on printed material? Would the design of the intervention be sufficiently flexible to accommodate such types of preferences? Will the participants be informed about the extent of their influence on delivery and design?

 Learning from the experience of engaging with older adults, I advise discussing such matters beforehand with the research team.

 P. 7: Figure 2. Primary outcomes: "Improved sleep quality and quality(?)"

 P. 7: Figure 2. How does “collaborative co-design” address “relatedness” for those future end users who have not been part of the development? The SDT is used as a behaviour change model, which “can be applied to optimise light exposure among older adults and promote sustainable behaviour change in this population”. But “relatedness”, as described by the authors, refers to the developmental phase, not the use phase. I can think of some examples of “relatedness” in the use phase, such as in-person meetings, a community chat group for exchanging ideas about routines to increase daily light exposure or encouraging joint walks outdoors.

 P. 9-10: A clarification is needed. Is the outcome after workstream 1 an early prototype of a smartphone application? It would be good to make it explicit in Figure 2 (the prototype is not mentioned here until after Workstream 2). Or is it the “Minimum Viable Product (MVP) version of the LightSPAN” first described in workstream 2?

 P. 10: How will the community service providers be selected (any inclusion criteria?)? How will their feedback on the early prototype be collected - through workshops, focus groups, individual interviews, or other collection techniques? The study sample, including the older adults, is described in detail, unlike the sample which includes the community service providers.

 P. 10: Study sample: Why will the sample, including 20 participants, be divided into four groups? Is it because there will be five focus groups with five participants in each group?

 P. 13: The purpose of the telephone interviews with 20 older adults is to understand better their current light exposure behaviour and preferences regarding health and technology. How exactly will the findings inform the subsequent stages of the study and the development of the intervention (“serve as a platform”)? Is there an iteration missing in Figure 1? Or will the findings from the telephone interviews guide the development of the focus group interview guide?

 On page 18 (Discussion), the authors provide a much more apparent reason for including the telephone interviews: “We assess participants’ baseline technological and health literacy levels during the telephone interview stage to determine support requirements in conducting discussions with them.”

 P. 13: The focus group discussion aims to get feedback on user perceptions and attitudes, feedback on the LightSPAN mHealth intervention plan, and feature preferences. How many participants are there in each focus group? I assume not all 20 participants take part in one focus group.

 P. 15-16: Check the placement of Tables according to the in-text reference to, for example, Table 7.

 P. 17: How many will take part in the prototyping workshop when the participants evaluate and discuss a developed version of the prototype? How many workshops? I assume it is the same sample as in the telephone interviews and the focus group discussion.

 P. 17: Outputs: “The current study will yield a co-designed app and intervention program meticulously tailored to the needs of older adults, which will then be carried onto a randomised controlled trial (RCT) for further evaluation.”

 In the discussion, the authors state that the intervention will be pilot-tested as part of a final and third workstream (not included in the current protocol). “A pilot study is planned to evaluate the feasibility of the LightSPAN intervention before broader implementation.”

 I suggest replacing the “randomised controlled trial (RCT)” with “a pilot study evaluating the feasibility of the intervention”.

 P. 17: Outputs: “co-designed app and intervention program...” Some clarification is needed. Is not the intervention delivered as a mobile phone app? Or might I have missed any other intervention elements?

 P. 18: The authors can decide whether “co-design” is necessary. The conclusion (after deleting “co-design”) still reflects a user-centred approach and an empathic design approach.

Is the study design appropriate for the research question?

Yes

Is the rationale for, and objectives of, the study clearly described?

Yes

Are sufficient details of the methods provided to allow replication by others?

Partly

Are the datasets clearly presented in a useable and accessible format?

Not applicable

Reviewer Expertise:

Environmental psychology, applied gerontology, and lgiht and health

I confirm that I have read this submission and believe that I have an appropriate level of expertise to confirm that it is of an acceptable scientific standard, however I have significant reservations, as outlined above.

# Response

**Published:** 2025-06-13

**Authors:** Manuel Spitschan

**Affiliations:** Not Applicable, Germany

**R1.1**

**Drawing on the title (co-design), I expected more active collaboration with stakeholders and target users throughout the study – from start to end. I would characterise the study as a two-part (or two-phase) development of a health-promoting intervention. The intervention targets light exposure among older adults. The first phase (referred to as Workstream 1 by the authors) consists of foundational activities resulting in an early prototype of a mobile phone application, which is followed by a second stage (Workstream 2), including continued development and evaluation by future community service providers and target users (community-dwelling older adults, age 60 and over). Thus, a co-design approach will be applied in the second phase. The intervention – delivered via a mobile app – will be refined between the two phases and within the second.**

**I do not suggest any changes to the protocol but rather an explication of what the authors mean by a co-design approach (to justify the title), minor elaborations on some procedure details and that the Background section is shortened to make space for an extended paragraph on co-design, situating the current study in more detail in the area of co-design, (e.g., addressing the frequency of co-designers’ involvement, timing – where in the process – and influence on the design solutions).**

 We now explain the co-design approach in the section ‘Co-design approach for the development of healthy ageing mHealth interventions” in the background.

**R1.2**

**I look forward to reading the results when the study is completed. Light-related interventions directed at older adults are much needed.**

 Thank you for your support. We are excited about the study as well.

**R1.3**

**Below are some questions asking for clarification or suggestions, hoping they will improve the manuscript before indexing.**

**Abstract: “open-ended discussions”. Could you be more specific – workshops, group interviews, focus group discussions?**

 We have clarified this to say “focus group discussion’.

**R1.4**

**Abstract (the final sentence): “... will ensure that the intervention addresses the needs of its future users.” Consider rephrasing to, for example: “facilitate future implementation”. You will probably not capture the needs of all future users (considering the rapid development of technology). However, a limited number of participants will certainly help to capture many potential issues.**

 We have updated the final sentence in the abstract following the reviewer’ suggestion.

**R1.5**

**P. 3: (subtitle) “ageing health” –> “healthy ageing”. Also, this subsection could be shorter. A background section should not be overly long in a study protocol.**

 We have corrected the section title. We would like to leave the section as is, as it highlights the biological mechanisms that are affected in age, and which we aim to target through our light-based intervention.

**R1.6**

**P. 3: “Exposure to bright light, particularly natural sunlight, ...” Sunlight or daylight?**

 The intended concept was daylight, which we have now corrected.

**R1.7**

**P. 3: “To address this, ...” Challenges, issues?**

 We have now rephrased this as “To address these age-related challenges, …”.

**R1.8**

**P. 4: “Overall, studies co-designing technologies for health management, fitness, or in-home wellness have recognised the importance of including older adults as co-designers.” Missing word: “studies of”?**

 We have now rephrased this as “Overall, studies using a co-design approach for technologies in health management, fitness, or in-home wellness have recognised the importance of including older adults as co-designers”.

**R1.9**

**P. 4: “ensuring maximum feasibility and sustainability.” Word choice – maybe more cautious – “facilitating” or “promoting”. Considering the rapid technology development and often long co-design processes, it might be better to be a bit cautious.**

 We agree with the reviewer and have switched the expression “maximum feasibility and sustainability” with “facilitating feasibility and sustainability”

**R1.10**

**P. 4: Word choice: “urging them”. Maybe “supporting” or “encouraging them”?**

 We have rephrased this to say “… supporting them in adopting …”

**R1.11**

**P. 4: “The intervention utilises the LightSPAN mHealth app...” Confusing – is the app already developed? Or should it be rephrased to “will be provided using the ...”**

 We have rephrased this to say “The intervention will be delivered through a novel app, the LightSPAN mHealth app, …”.

**R1.12**

**P. 4: “... the potential efficacy of a technology-driven, lifestyle-oriented intervention ...”. There is a misalignment here in giving technology priority. Your intention is to develop a user-centred intervention to address lifestyle issues (“... the potential efficacy of a lifestyle-oriented intervention using smartphone technology...”).**

 We have rephrased this to say “…a lifestyle-oriented intervention using smartphone/app …”.

**R1.13**

**P. 5: “mHealth user personas, ...”. Using personas is a helpful initial tool in the early step of development until you have conducted the interviews with the target users. Have you also considered using the interview findings in the next step (workstream 2) to create project-specific personas? Personas based on your interviews might be more helpful for further developing the intervention than more generic user profiles.**

 We thank the reviewer for this suggestion. It would be an interesting exercise to map the findings for interviews to personas, we will postpone this additional analysis until the main trial, from which we hope not only to identify users, but use the quantitative light exposure data to map different ‘light exposure (behaviour) types’.

**R1.14**

**P. 5 and 6: The workstreams described on page 5 do not entirely reflect Figure 1 on page 6. For example, is the prototype developed before workstream 2 (as shown in Figure 1, "initial development of LightSPAN mHealth app), or is it included in workstream 2 as the workstream title indicates on page 5 ("Co-design o[f] the mHealth intervention")?**

 We have revised the text and Figure 1 to improve clarity and better align with the described process:

 Initial development has been rephrased to clearly indicate that it is integration of evidence and insights to inform intervention design The prototype will not be finalized before Workstream 2. Rather, it is developed iteratively throughout the activities in Workstream 2b through older adult engagement across telephone interviews, focus group discussions, and prototyping workshops.

**R1.15**

**Also, I find new terms in Figure 1 a bit confusing, such as "LightSPAN Behaviour Change Model" and "infographic with recommendations for mHealth design".**

 We have clarified the terminology in Figure 1:

 "LightSPAN Behaviour Change Model" now clearly refers to the application of SDT principles as the working framework for the intervention.

 "Infographic with recommendations" has been revised to "Synthesis of recommendations for mHealth app design" to better reflect the structured output rather than a visual product.

**R1.16**

**In Figure 1, a LightSPAN mHealth prototype app is developed after engaging with community service providers but before engaging with older adults.**

 This represents the point where we began developing the prototype after engaging with community service providers and consolidating their feedback. We then continued building and refining the prototype iteratively with older adult participants throughout Workstream 2. The figure has been amended for better clarity of the process.

**R1.17**

**The unclarities mentioned above make me wonder whether this is a co-design project or a user-centred development of intervention, including need assessments and user evaluations.**

 This is a co-design project.

**R1.18**

**Relating to this, in the conclusion on page 5, the authors write: "A co-design approach will be employed to develop an effective mHealth intervention tailored to our target users, involving active collaboration between the researchers and older adult participants." But Figure 1 shows something slightly different: you will develop an early prototype – get feedback from the community service provider – refine the prototype - and then you will ask older adults about their needs and preferences – and finally get feedback on the prototype by older adults participating in focus groups. However, the procedure described in the study might be regarded as a co-design study in a weaker sense. Then, the authors could describe their definition of a co-design approach.**

 The co-design process engages older adult participants in multiple stages, including needs assessment (telephone interviews), ideation and discussion (focus group discussions), and hands-on feedback and testing (prototyping workshops). At each stage, feedback will be used to iteratively refine the app prototype.

**R1.19**

**P. 6: Co-design team. The co-design team, as named by the authors, includes researchers and a coordinator, but no stakeholders or target users as expected. Why not refer to the team as a research team?**

 We have switched the term to now be “research project team”.

**R1.20**

**P. 6: "The team will be assigned roles ..." Do you mean during the workshop and focus groups? Will all team members participate? What is the difference between a moderator and a facilitator?**

 Yes, the roles of moderator, facilitator, and notetaker refer to responsibilities assigned during both the workshop and focus group discussions. Designated team members will take on one of the assigned roles which are now outlined. We have revised the text.

**R1.21**

**P. 6: Guiding principles. The principles are well described and essential to the intervention's development. To achieve the design objectives, the authors highlight, for example, "To facilitate this, tailored interventions must be delivered appropriately for older adults, ..."**

**I wonder if you have considered how you will manage input from the stakeholders or end users concerning intervention delivery. An app-based solution may not be ideal. What if they express preferences for in-person meetings? Or getting tech support (on more occasions than at the introductory session)? Or reading the intervention content on printed material? Would the design of the intervention be sufficiently flexible to accommodate such types of preferences? Will the participants be informed about the extent of their influence on delivery and design?**

**Learning from the experience of engaging with older adults, I advise discussing such matters beforehand with the research team.**

 This is an excellent point. The intervention itself is designed to be linked to a light measurement device, which is worn by the participant. As a consequence, to be able to operate on these data, a system for feedback of information on exposure is needed. It is very hard to conceive what this might look like without a smartphone. Additionally, implicit in this work is the possibility of a scalable intervention, which is achievable through smartphones. In the “Guiding principles” section, we have added the following sentence: “A core concept in the delivery of the intervention, the deployment of an app, allows for the scalabibility of the approach.”

**R1.22**

**P. 7: Figure 2. Primary outcomes: "Improved sleep quality and quality(?)"**

 Thank you for pointing that out. The figure has been revised to correct the typographical error, and “sleep quality and quantity” is now accurately reflected.

**R1.23**

**P. 7: Figure 2. How does “collaborative co-design” address “relatedness” for those future end users who have not been part of the development? The SDT is used as a behaviour change model, which “can be applied to optimise light exposure among older adults and promote sustainable behaviour change in this population”. But “relatedness”, as described by the authors, refers to the developmental phase, not the use phase. I can think of some examples of “relatedness” in the use phase, such as in-person meetings, a community chat group for exchanging ideas about routines to increase daily light exposure or encouraging joint walks outdoors.**

 Future iterations of the intervention can include this concept, e.g., through "light meet-ups" and leaderboards.

**R1.24**

**P. 9-10: A clarification is needed. Is the outcome after workstream 1 an early prototype of a smartphone application? It would be good to make it explicit in Figure 2 (the prototype is not mentioned here until after Workstream 2). Or is it the “Minimum Viable Product (MVP) version of the LightSPAN” first described in workstream 2?**

 Workstream 1 does not include the development of an app; this follows in Workstream 2, which we have clarified now: “This process informs the intervention design, focusing on developing the LightSPAN mHealth app developed following the additional work in Workstream 2.” Figure 2 is designed to given an insight into the working model of the underlying behavioural mechanisms, and does not reflect the process.

**R1.25**

**P. 10: How will the community service providers be selected (any inclusion criteria?)? How will their feedback on the early prototype be collected - through workshops, focus groups, individual interviews, or other collection techniques? The study sample, including the older adults, is described in detail, unlike the sample which includes the community service providers.**

 We have now clarified this section to say: “Invitations will be extended to selected community service providers working at Lions Befrienders (LB), a social service agency in Singapore dedicated to providing care and support for older adults. We will sample based on convenience, with no specific inclusion or exclusion criteria.”

**R1.26**

**P. 10: Study sample: Why will the sample, including 20 participants, be divided into four groups? Is it because there will be five focus groups with five participants in each group?**

 Yes, participants will be divided into four groups of five to allow for manageable group sizes and in-depth engagement for the focus group discussion and prototyping workshops.

**R1.27**

**P. 13: The purpose of the telephone interviews with 20 older adults is to understand better their current light exposure behaviour and preferences regarding health and technology. How exactly will the findings inform the subsequent stages of the study and the development of the intervention (“serve as a platform”)? Is there an iteration missing in Figure 1? Or will the findings from the telephone interviews guide the development of the focus group interview guide?**

**On page 18 (Discussion), the authors provide a much more apparent reason for including the telephone interviews: “We assess participants’ baseline technological and health literacy levels during the telephone interview stage to determine support requirements in conducting discussions with them.”**

 We have now moved this key piece of information up, and placed in the “Telephone interview” section: “We assess participants’ baseline technological and health literacy levels during the telephone interview stage to determine support requirements in conducting discussions with them.”

**R1.28**

**P. 13: The focus group discussion aims to get feedback on user perceptions and attitudes, feedback on the LightSPAN mHealth intervention plan, and feature preferences. How many participants are there in each focus group? I assume not all 20 participants take part in one focus group.**

 We have clarified this and added the following sentence in the Procedure: “Sessions will include up to five participants.”

**R1.29**

**P. 15-16: Check the placement of Tables according to the in-text reference to, for example, Table 7.**

 We believe that in the final manuscript version, it has been placed correctly, and will ensure this in the proofing process..

**R1.30**

**P. 17: How many will take part in the prototyping workshop when the participants evaluate and discuss a developed version of the prototype? How many workshops? I assume it is the same sample as in the telephone interviews and the focus group discussion.**

 Yes, the same sample of 20 participants will take part in all three activities: telephone interviews, focus group discussions (FGDs), and prototyping workshop (PW) sessions. The participants will be divided into four groups of five, and each group will take part in one FGD and one prototyping workshop.

 We have clarified in the revised text.

**R1.31**

**P. 17: Outputs: “The current study will yield a co-designed app and intervention program meticulously tailored to the needs of older adults, which will then be carried onto a randomised controlled trial (RCT) for further evaluation.” In the discussion, the authors state that the intervention will be pilot-tested as part of a final and third workstream (not included in the current protocol). “A pilot study is planned to evaluate the feasibility of the LightSPAN intervention before broader implementation.” I suggest replacing the “randomised controlled trial (RCT)” with “a pilot study evaluating the feasibility of the intervention”.**

 We will in fact run two additional trials following on from the app: a pilot and feasibility trial, and an RCT. We have now clarified this in the text: “The current study will yield a co-designed app-delivered intervention program tailored to the needs of older adults, which will then be carried onto two additional trials: a pilot and feasibility trial and a randomised controlled trial (RCT) for further evaluation.”

**R1.32**

**P. 17: Outputs: “co-designed app and intervention program...” Some clarification is needed. Is not the intervention delivered as a mobile phone app? Or might I have missed any other intervention elements?**

 We understand the confusion. We have now clarified this to say “co-designed app-delivered intervention program”

**R1.33**

**P. 18: The authors can decide whether “co-design” is necessary. The conclusion (after deleting “co-design”) still reflects a user-centred approach and an empathic design approach.**

 We thank the reviewer for their careful review.
